# Outcome of Community-Acquired *Staphylococcus aureus* Bacteraemia in Patients with Diabetes: A Historical Population-Based Cohort Study

**DOI:** 10.1371/journal.pone.0153766

**Published:** 2016-04-15

**Authors:** Jesper Smit, Reimar Wernich Thomsen, Henrik Carl Schønheyder, Henrik Nielsen, Trine Frøslev, Mette Søgaard

**Affiliations:** 1 Department of Clinical Microbiology, Aalborg University Hospital, Aalborg, Denmark; 2 Department of Infectious Diseases, Aalborg University Hospital, Aalborg, Denmark; 3 Department of Clinical Epidemiology, Aarhus University Hospital, Aarhus, Denmark; 4 Department of Clinical Medicine, Aalborg University, Aalborg, Denmark; Osaka University Graduate School of Medicine, JAPAN

## Abstract

**Background:**

Patients with diabetes (DM) experience increased risk of *Staphylococcus aureus* bacteraemia (SAB), but the prognostic impact of diabetes in patients with SAB remain unclear. Therefore, we investigated 30-day all-cause mortality in patients with and without DM.

**Methods:**

Population-based medical databases were used to conduct a cohort study of all adult patients with community-acquired SAB in Northern Denmark, 2000–2011. Using Cox proportional hazards regression, we computed hazard ratios as estimates of 30-day mortality rate ratios (MRRs) among patients with and without DM. We further investigated whether the prognostic impact of DM differed among patients with and without recent preadmission healthcare contacts (within 30 days of the current hospitalization) and by age, sex, marital status, level of comorbidity, and DM-related characteristics (e.g., duration of DM and presence of DM complications).

**Results:**

Among 2638 SAB patients, 713 (27.0%) had DM. Thirty-day cumulative mortality was 25.8% in patients with DM and 24.3% in patients without DM, for an adjusted MRR (aMRR) of 1.01 (95% confidence interval (CI), 0.84–1.20). In analyses with and without recent healthcare contacts, the corresponding aMRRs were 0.84 (95% CI, 0.62–1.14) and 1.13 (95% CI, 0.91–1.41), respectively. Compared to patients without DM, the aMRR was 0.94 (95% CI, 0.74–1.20) for male patients with DM and 1.13 (95% CI, 0.87–1.47) for female patients with DM. The prognostic influence of DM on mortality did not differ notably with age, level of comorbidity, or characteristics of patients with DM.

**Conclusion:**

Patients with DM and community-acquired SAB did not experience higher 30-day mortality than patients without DM.

## Introduction

*Staphylococcus aureus* is a leading cause of bacteraemia, with a 30-day mortality of 20–40% in developed countries [1–5]. Diabetes mellitus (DM) is associated with considerable morbidity and mortality, and the prevalence of this chronic disease is rapidly increasing on a global scale [6–7]. Patients with DM may experience higher mortality from *S*. *aureus* bacteraemia (SAB) than patients without DM because of generally decreased immunity [8], potential diabetes complications including renal disease, and a high prevalence of shared negative prognostic factors of SAB including advanced age and comorbidity [3].

Data regarding the prognostic impact of DM in patients with SAB remain sparse and conflicting, however, and to our knowledge, no study has examined this impact of DM as a primary objective. Previous investigations have been limited by small numbers of patients with DM, use of non-validated algorithms to identify DM patients, and failure to incorporate concurrent chronic conditions [9–14]. We find no studies that have assessed whether the prognostic influence of DM on SAB differs across gender, age categories, or comorbidity levels, and DM has been treated as one entity disregarding duration of disease, quality of glycaemic control, and complications. Moreover, only one of these studies [11] has differentiated SAB with recent preadmission healthcare exposure (healthcare-associated (HCA)-SAB) from patients without it, although the two patient groups have been suggested to differ considerably with regard to prognosis [15–17].

Detailed data including complete follow-up are needed to clarify whether DM is associated with increased mortality in patients with SAB. This knowledge may extend our understanding of the clinical course of patients with SAB, help define high-risk groups to assist in risk stratification and patient triage, and contribute to overall optimized care for patients with DM. Therefore, we conducted a large historical population-based cohort study to elucidate 30-day all-cause mortality in patients with community-acquired SAB (CA-SAB), comparing patients with and without DM. We ascertained the prognostic impact of DM on mortality among patients with and without recent preadmission healthcare contact, and stratified by age, sex, marital status, and comorbidity level. Finally, we explored 30-day mortality in SAB patients according to characteristics of patients with DM (e.g., duration of DM, quality of glycaemic control, renal function, and presence of DM complications).

## Patients and Methods

### Setting

This historical cohort study was conducted using routinely recorded data from population-based medical registries and databases in Northern Denmark between January 1, 2000, and December 31, 2011 (catchment population ~ 1.8 million). Tax-supported, unrestricted healthcare is provided for the entire Danish population through a national insurance program. All Danish citizens are assigned a unique identification number (the civil registration number) upon birth or immigration, which allows unambiguous cross-linkage of records between the data sources [18–19].

### Patients with *S*.*aureus* bacteraemia

We identified all patients hospitalized with CA-SAB in the databases of the regions’ four departments of clinical microbiology from 1995 onwards. Identification and susceptibility testing of *S*. *aureus* isolates are described in S1 Appendix. Eligible cases were defined by the presence of ≥1 monomicrobial positive blood culture with *S*. *aureus* in patients aged ≥15 years. Because recurrence of SAB may influence prognosis [20], we limited inclusion to patients with an incident episode of bacteraemia with *S*. *aureus*, defined as no prior SAB diagnosis within at least 5 years of the current hospitalization.

We defined CA-SAB as SAB in patients in whom one or more positive blood cultures was obtained ≤2 days of admission. Patients whose first blood culture was obtained >2 days after admission were excluded because we considered these infections to have been hospital-acquired. Patients with CA-SAB and healthcare contact within 30 days of the current admission were further sub-classified as HCA-SAB if one or more of the following criteria were fulfilled: one or more hospital admissions, one or more contacts with hospital outpatient surgical clinics, or one or more contacts with hospital outpatient clinics of oncology, haematology, or nephrology [21].

Data on recent healthcare contacts were obtained from the Danish National Patient Registry (DNPR) [22–23], which holds data on all citizens admitted to Danish hospitals since 1977 and on outpatient clinic visits since 1995. For each contact, data include dates of hospital admission and discharge, up to 20 discharge diagnoses, and information on surgical procedures.

### Patients with diabetes

Patients with DM were diagnosed prior to admission and identified using a validated three-step algorithm [24]. First, patients with a discharge or outpatient diagnosis of DM registered at any time prior to admission were identified in the DNPR. Second, patients with a glycosylated haemoglobin A1c (HbA1c) level diagnostic of DM (≥48 mmol/mol or ≥6.5%) measured at any time predating the admission were identified via the clinical laboratory information system (LABKA) research database [25]. The LABKA database contains information on specimens submitted for analysis by hospitals and practitioners in Northern Denmark for the entire study period, including the exact time of blood sample collection. Third, the Aarhus University Prescription Database [26] (AUPD) contains data on all filled prescriptions in the study area according to the anatomical therapeutic chemical (ATC) classification system. Using this register, we identified patients with at least one recorded prescription for any glucose-lowering medication at any time prior to the current hospitalization (for diagnostic, laboratory, and ATC codes, see S2 Appendix). Patients with DM were classified as either type 1 diabetes (persons diagnosed before age 30 years, using insulin monotherapy, and with no history of oral glucose-lowering medications) or type 2 diabetes (the remaining patients with DM).

### Characteristics of patients with diabetes

Duration of DM was computed as the time elapsed between the first record of diabetes in any of the three registers and the date that the first positive blood culture was sampled. Data on diabetes micro- and macrovascular complications were obtained from the DNPR [22–23] (for diagnostic codes, see S2 Appendix). The level of glycaemic control was ascertained using the most recent HbA1c measurement within one year of the current hospitalization (available for 70% of the patients), and we obtained data on blood glucose level on the date of admission (available for 55% of the patients). To assess baseline renal function prior to admission, we retrieved the most recent creatinine measurement requested by an outpatient hospital clinic or general practitioner in the period from one year to seven days before the current admission (available for 78% of the patients). Estimated glomerular filtration rates (eGFR) were calculated using the four-variable version of the Modification of Diet in Renal Disease equation [27] (equation provided in S2 Appendix).

### Demographics, comorbidity, and medication

We obtained data on age, sex, and marital status from the Danish Civil Registration System, which is electronically updated daily and holds records of demographic data and all changes in vital status and migration for the entire Danish population since 1968 [18–19].

We used all diagnoses recorded in the DNPR up to 10 years prior to the current admission date to identify previous comorbidity included in the Charlson Comorbidity Index (CCI). The CCI is a validated comorbidity scoring system [28–29] covering the number and severity of 19 major disease categories. Because DM constituted the exposure variable of interest, we removed this condition from the CCI and designated the index as a modified CCI (m-CCI). Three levels of comorbidity were defined: “low” (0), corresponding to patients with no pre-existing registered comorbidity; “intermediate” (1–2); and “high” (>2). Furthermore, we obtained data on several factors not registered in the m-CCI, including alcohol- and drug-related diagnoses, hypertension, and dialysis (within 30 days of the current admission) (for diagnostic codes, see S2 Appendix). Because certain medications might influence SAB prognosis via immunomodulatory effects [30], we also retrieved information on the following filled prescriptions via the AUPD[24]: any previous use of antihypertensive treatment, statins, anticoagulants, and use of immunosuppressant drugs and antibiotic treatment within 30 days of the admission (for ATC codes, see S2 Appendix). We assessed all-cause 30-day mortality for patients with and without DM using the Danish Civil Registration System [18–19].

### Statistical analyses

Follow-up was initiated on the date the first positive blood culture was drawn, and vital status was followed until death, migration, or for 30 days, whichever came first. Patient characteristics were tabulated according to diabetes status. Using the Kaplan–Meier method, we computed mortality function curves (1 –survival function) and cumulative mortality at 30 days for patients with and without DM. We compared 30-day mortality rates for patients with and without DM using a Cox proportional hazards model to estimate hazard ratios as a measure of mortality rate ratios (MMRs) with corresponding 95% confidence intervals (CIs). Because the influence of DM on mortality may differ among patients with and without preadmission healthcare contacts [15–17], we repeated all analyses with restriction of the study cohort alternately to patients with CA-SAB and HCA-SAB, respectively. To ascertain the potential differential impact of DM in subgroups of patients, we stratified the analyses by gender, age category (15–39, 40–59, 60–79, 80+ years), marital status (married, divorced or widowed, never married), and m-CCI level (“low”, “intermediate”, and “high”). We adjusted for age, gender, m-CCI score, hypertension, alcohol-related conditions, marital status, and preadmission use of statins and antibiotics. In a subgroup analysis limited to patients with DM, we ascertained 30-day mortality according to duration of DM, level of glycaemic control, presence of DM complications, level of glucose on admission, and baseline preadmission renal function.

All Cox regression analyses were preceded by a graphical verification of the proportional hazards assumption. The statistical analyses were performed using Stata 11.2 for Windows (Stata Corp, College Station, TX). According to Danish law, individual informed consent is not required for observational registry-based studies. The project was approved by the Danish Data Protection Agency (ref. no. 2012-41-0942). Data were not anonymized prior to analysis.

## Results

### Descriptive data

From 2000 to 2011, we identified 2638 patients with incident CA-SAB (Table 1), of whom 713 (27.0%) had DM.

**Table 1:** Characteristics of 2638 patients hospitalized with incident community-acquired *S*. *aureus* bacteraemia in Northern Denmark, 2000–2011.

	Patients with diabetes	Patients without diabetes
Numbers (%)	713 (27.0)	1925 (73.0)
Age (years), median (IQR)	71.1 (60.6–79.9)	68.1 (53.9–79.1)
15–39 years	27 (3.8)	206 (10.7)
40–59 years	143 (20.1)	462 (24.0)
60–79 years	367 (51.5)	815 (42.3)
≥80 years	176 (24.7)	442 (23.0)
Sex		
Men	452 (63.4)	1164 (60.5)
Women	261 (36.6)	761 (39.5)
*S*. *aureus* bacteraemia		
Community acquired	398 (55.8)	1125 (58.4)
Healthcare associated	315 (44.2)	800 (41.6)
MRSA	3 (0.4)	10 (0.5)
Marital status		
Married	343 (48.1)	927 (48.2)
Divorced or widowed	264 (37.0)	622 (32.3)
Never married	106 (14.9)	376 (19.5)
Selected comorbidities in the modified Charlson Comorbidity Index		
Former myocardial infarction	98 (13.7)	122 (6.3)
Congestive heart failure	164 (23.0)	184 (9.6)
Peripheral vascular disease	163 (22.9)	165 (8.6)
Cerebrovascular disease	116 (16.3)	199 (10.3)
Chronic pulmonary disease	118 (16.6)	245 (12.7)
Moderate to severe renal disease	184 (25.8)	252 (13.1)
Any solid cancer	101 (14.2)	414 (21.5)
Modified Charlson Comorbidity Index score		
Low (0)	156 (21.9)	654 (34.0)
Intermediate (1–2)	286 (40.1)	726 (37.7)
High (>2)	271 (38.0)	545 (28.3)
Comorbidities, other types		
Hypertension	296 (41.5)	355 (18.4)
Dialysis within 30 days of admission	111 (15.6)	153 (8.0)
Conditions related to alcohol abuse	67 (9.4)	168 (8.7)
Conditions related to drug abuse	10 (1.4)	63 (3.3)
Preadmission medication use		
Immunosuppressive therapy	6 (0.8)	22 (1.1)
Systemic antibiotic therapy	162 (22.7)	374 (19.4)
ACE inhibitors	493 (69.1)	593 (30.8)
Beta blockers	376 (52.7)	659 (34.2)
Acetylsalicylic acid	427 (60.0)	694 (36.1)
Statins	321 (45.0)	304 (15.8)
Renal function prior to admission		
eGFR >90 mL/min per 1.73 m^2^	90 (12.6)	279 (14.5)
eGFR 60–90 mL/min per 1.73 m^2^	155 (21.7)	450 (23.4)
eGFR <60 mL/min per 1.73 m^2^	311 (43.6)	491 (25.5)
eGFR missing	157 (22.0)	705 (36.6)
CRP on day of blood culture (mg/L), median (IQR)	171 (76–274)	177 (76–287)
<10 mg/L	11 (1.5)	36 (1.9)
10–100 mg/L	177 (24.8)	452 (23.5)
>100 mg/L	406 (56.9)	1088 (56.5)
CRP missing	119 (16.7)	349 (18.1)

Patients with type 1 diabetes = 5.9%.

IQR: interquartile range. MRSA: methicillin resistant *S*. *aureus*. ACE inhibitor: angiotensin-converting enzyme inhibitor. eGFR: estimated glomerular filtration rate. CRP: C-reactive protein.

There were slightly more men among patients with DM compared to patients without DM (63.4% vs. 60.5%). Median age was 71 and 68 years for patients with and without DM, respectively. Of the 2638 patients with SAB, 69% had one or more conditions registered in the m-CCI and patients with DM were more likely than those without DM to have a high m-CCI score. The proportion of patients classified as HCA-SAB did not differ notably between patients with and without DM (44% and 42%, respectively). Compared to patients without healthcare association (CA-SAB), patients with HCA-SAB had considerably more comorbidity registered in the m-CCI whereas patients with CA-SAB were older (median age 66 years vs. 72 years) and more frequently male (60% versus 63%).

### Thirty-day mortality

**Fig 1:**
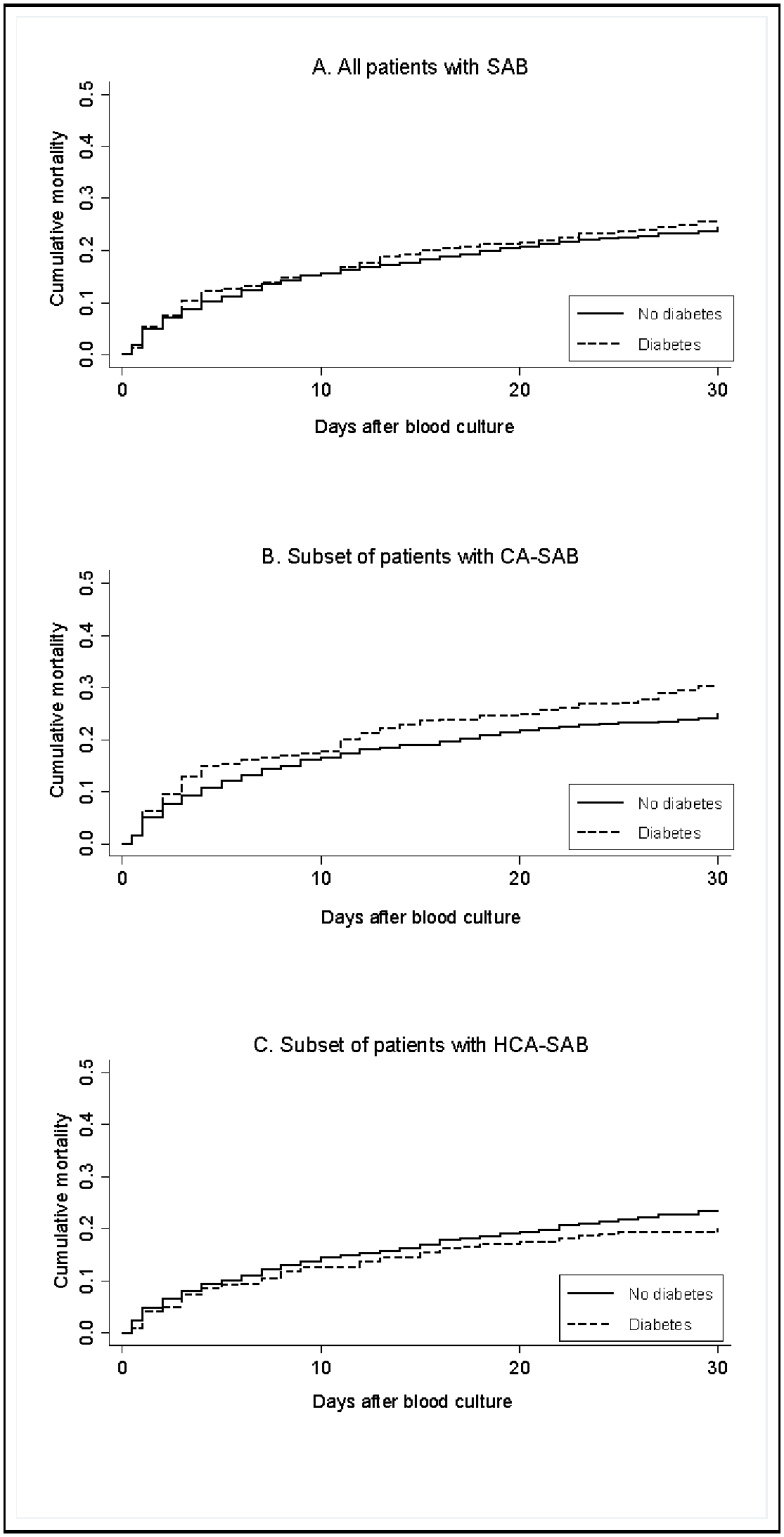
Cumulative 30-day mortality among incident *S*. *aureus* bacteraemia (SAB) patients with and without diabetes. (A) Cumulative 30-day mortality including the entire cohort of SAB patients (community-acquired SAB (CA-SAB) and healthcare-associated SAB (HCA-SAB)). (B) Cumulative 30-day mortality in the subset of patients with CA-SAB. (C) Cumulative mortality in the subset of patients with HCA-SAB.

Thirty-day cumulative mortality was 25.8% in patients with DM and 24.3% in patients without DM (Table 2 and Fig 1).

**Table 2:** Crude and adjusted 30-day mortality in patients with incident *S*. *aureus* bacteraemia (SAB).

	n	30-day mortality (95% CI)	MRR^1^ (95% CI)	aMRR^2^ (95% CI)
**All SAB**				
No diabetes	1925	24.3 (22.5–26.3)	1.00 (ref.)	1.00 (ref.)
Diabetes	713	25.8 (22.8–29.2)	1.07 (0.90–1.27)	1.01 (0.84–1.20)
Type 1 diabetes	40	5.0 (1.3–18.6)	0.19 (0.47–0.75)	0.59 (0.14–2.39)
Type 2 diabetes	673	27.0 (23.9–30.6)	1.13 (0.95–1.34)	1.01 (0.85–1.21)
**CA-SAB**				
No diabetes	1125	24.9 (22.5–27.5)	1.00 (ref.)	1.00 (ref.)
Diabetes	398	30.4 (26.1–35.2)	1.26 (1.02–1.56)	1.13 (0.91–1.41)
**HCA-SAB**				
No diabetes	800	23.5 (20.7–26.6)	1.00 (ref.)	1.00 (ref.)
Diabetes	315	20.0 (15.9–24.9)	0.84 (0.63–1.11)	0.84 (0.62–1.14)

CI: confidence interval. CA-SAB: community-acquired SAB. HCA-SAB: healthcare-associated SAB.

^1^MRR: unadjusted mortality rate ratio.

^2^aMRR: MRR adjusted for age, gender, marital status, conditions included in the modified Charlson Comorbidity Index, hypertension, alcohol-related conditions, any previous statin use prior to admission, and antibiotic therapy 30 days prior to admission.

Overall, patients with DM experienced no increased risk of dying within 30 days of blood culture compared to patients without DM (adjusted MRR (aMRR) = 1.01 (95% CI, 0.84–1.20). In corresponding analyses restricted to patients with CA-SAB and HCA-SAB, the aMRRs were 1.13 (95% CI, 0.91–1.41) and 0.84 (95% CI, 0.62–1.14), respectively. Table 3 displays the prognostic impact of DM across gender, age groups, marital status, and level of comorbidity.

**Table 3:** Adjusted mortality within 30 days comparing incident community-acquired *S*. *aureus* bacteraemia in patients with and without diabetes, stratified by sex, age, marital status, and modified Charlson Comorbidity Index score.

	Patients without diabetes	Patients with diabetes	
	30-day mortality (95% CI)	30-day mortality (95% CI)	aMRR^1^ (95% CI)
Overall	24.3 (22.5–26.3)	25.8 (22.8–29.2)	1.00 (0.84–1.20)
Sex			
Male	22.0 (19.7–24.5)	21.9 (18.4–26.0)	0.94 (0.74–1.20)
Female	27.9 (24.8–31.2)	32.6 (27.3–38.6)	1.13 (0.87–1.47)
Age			
15–39	2.9 (1.3–6.4)	3.7 (0.5–23.5)	4.69 (0.47–46.41)
40–59	13.9 (11.0–17.4)	12.6 (8.1–19.2)	1.18 (0.66–2.10)
60–79	26.0 (23.1–29.2)	23.7 (19.7–28.4)	0.89 (0.68–1.16)
80+	42.1 (37.6–46.8)	44.3 (37.3–52.0)	1.12 (0.86–1.48)
Marital status			
Married	20.9 (18.5–23.7)	21.9 (17.9–26.6)	1.02 (0.77–1.35)
Divorced or widowed	34.7 (31.1–38.6)	31.8 (26.6–37.8)	0.93 (0.71–1.20)
Never married	15.4 (12.2–19.5)	23.6 (16.6–32.9)	1.42 (0.84–2.40)
Modified Charlson Comorbidity Index score			
Low (0)	19.3 (16.4–22.5)	19.2 (13.9–26.3)	0.93 (0.61–1.40)
Intermediate (1–2)	24.7 (21.7–28.0)	26.2 (21.5–31.7)	1.04 (0.79–1.38)
High (>2)	29.9 (26.3–33.9)	29.2 (24.1–35.0)	1.02 (0.76–1.36)

Reference group: patients without diabetes. CI: confidence interval.

^1^aMRR: Mortality rate ratio adjusted for age, gender, marital status, conditions included in the modified Charlson Comorbidity Index, hypertension, alcohol-related conditions, any previous statin use prior to admission, and antibiotic therapy 30 days prior to admission.

Compared to patients without DM, the aMRRs were 0.94 (95% CI, 0.74–1.20) for male patients with DM and 1.13 (95% CI, 0.87–1.47) for female patients with DM. Age did not affect 30-day mortality; compared to patients without DM in the same age group, the aMRR values were as follows: 4.69 (95% CI, 0.47–46.41) for patients aged 15–39 years; 1.18 (95% CI, 0.66–2.10) for patients aged 40–59 years; 0.89 (95% CI, 0.68–1.16) for patients aged 60–79 years; and 1.12 (95% CI, 0.86–1.48) for patients ≥80 years. Analyses restricted to patients with CA-SAB (see S1 Table) and to patients with HCA-SAB (data not shown) revealed comparable results.

### Thirty-day mortality according to characteristics of patients with diabetes

Table 4 shows cumulative and relative 30-day mortality for patients with DM (n = 713) according to characteristics of patients with diabetes.

**Table 4:** Thirty-day mortality in 713 patients with diabetes and incident community-acquired *S*. *aureus* bacteraemia according to characteristics of patients with diabetes.

	n	30-day mortality (95% CI)	MRR^1^ (95% CI)	aMRR^2^ (95% CI)
Duration of diabetes				
0–2 years	176	29.0 (22.9–36.3)	1.00 (ref.)	1.00 (ref.)
3–5 years	144	24.3 (18.1–32.2)	0.79 (0.51–1.20)	0.72 (0.47–1.12)
6–10 years	123	26.0 (19.2–34.7)	0.84 (0.54–1.31)	0.77 (0.49–1.21)
>10 years	270	24.4 (19.8–30.0)	0.81 (0.56–1.16)	0.87 (0.59–1.27)
Hba1c				
<7%	243	26.3 (21.2–32.3)	1.00 (ref.)	1.00 (ref.)
≥7–<8%	94	34.0 (25.4–44.6)	1.35 (0.88–2.06)	1.71 (1.10–2.65)
≥8–<9%	72	18.1 (10.9–29.1)	0.66 (0.37–1.20)	0.93 (0.51–1.69)
≥9%	93	20.4 (13.6–30.1)	0.74 (0.44–1.23)	1.01 (0.60–1.71)
Unknown	211	26.5 (21.1–33.1)	1.01 (0.70–1.44)	1.14 (0.78–1.65)
Diabetes complications				
Absent	257	27.6 (22.6–33.5)	1.00 (ref.)	1.00 (ref.)
Microvascular	96	17.7 (11.4–26.9)	0.62 (0.36–1.05)	0.99 (0.56–1.72)
Macrovascular	215	30.2 (24.6–36.9)	1.11 (0.80–1.57)	1.04 (0.59–1.84)
Micro/macrovascular	145	21.4 (15.6–29.0)	0.74 (0.48–1.12)	0.98 (0.56–1.71)
Glucose on admission				
5–10 mmol/L	133	28.6 (21.7–37.1)	1.00 (ref.)	1.00 (ref.)
10–15 mmol/L	122	31.1 (23.7–40.2)	1.16 (0.74–1.81)	1.39 (0.88–2.19)
15–20 mmol/L	67	20.9 (13.0–32.7)	0.70 (0.38–1.29)	0.89 (0.48–1.66)
>20 mmol/L	58	29.3 (19.4–42.8)	1.08 (0.61–1.92)	1.37 (0.77–2.43)
Unknown	321	22.4 (18.3–27.4)	0.77 (0.52–1.14)	0.86 (0.56–1.27)
Renal function prior to admission (eGFR)				
>90 mL/min per 1.73 m^2^	90	22.2 (15.0–32.3)	1.00 (ref.)	1.00 (ref.)
60–90 mL/min per 1.73 m^2^	155	27.1 (20.8–34.8)	1.24 (0.73–2.11)	1.03 (0.58–1.81)
<60 mL/min per 1.73 m^2^	311	25.1 (20.6–30.3)	1.11 (0.68–1.82)	0.95 (0.56–1.61)
eGFR missing	157	28.0 (21.7–35.8)	1.28 (0.76–2.17)	1.13 (0.64–1.98)

CI: confidence interval. HbA1c: Haemoglobin A1c, using the most recent measurement within one year of the current hospitalization. eGFR: estimated glomerular filtration rate (mL/min per 1.73m^2^).

^1^MRR: unadjusted mortality rate ratio.

^2^aMRR: MRR adjusted for age, gender, marital status, conditions included in the modified Charlson Comorbidity Index (excluding the morbidity in question), hypertension, any previous statin use prior to admission, and antibiotic therapy 30 days prior to admission.

Duration of DM did not seem to influence 30-day mortality. Compared with 0–3 years of DM duration, the aMRRs were 0.72 (0.47–1.12) for 3–5 years of DM duration, 0.77 (95% CI, 0.49–1.21) for 6–10 years, and 0.87 (95% CI, 0.59–1.27) for >10 years. The presence of micro- or macrovascular complications did not affect 30-day mortality: Compared with DM patients without complications, the aMRRs were 0.99 (95% CI, 0.56–1.72) for patients with microvascular complications and 1.04 (0.59–1.84) for those with macrovascular complications, respectively. Likewise, no consistent pattern or major differences in mortality were observed according to the level of glycaemic control, glucose level on admission, and renal function prior to admission. Restricting the analyses to CA-SAB or HCA-SAB did not substantially change these results (data not shown).

## Discussion

In this cohort study of 2638 patients, we observed substantial mortality associated with incident CA-SAB, but patients with DM did not experience higher 30-day mortality than patients without DM. Patients with and without recent healthcare exposure did not differ substantially in terms of prognosis, and we observed no considerable differences in 30-day mortality related to gender, age group, marital status, or comorbidity level in patients with and without DM. Other factors with no effect on mortality were duration of DM, presence of micro- and macrovascular complications, renal function, and other characteristics of patients with diabetes.

A few studies on SAB outcome have included DM among several covariates in the prognostic models [9–14]. Our study supports results from a large pooled analysis of five observational cohort studies including 3395 adult patients with SAB [14]. In that analysis, the investigators identified an adjusted 30-day hazard ratio of 1.12 (95% CI, 0.95–1.33) and a corresponding 90-day estimate of 1.03 (95% CI, 0.88–1.19) comparing patients with and without DM. Our findings are also in accordance with a Swiss cohort study [13] reporting no association between DM and in-hospital mortality in 308 SAB patients from a single tertiary-care centre.

In contrast to our findings, however, three studies [9–10,31] have reported that DM is associated with increased mortality in patients with SAB. A US cohort study [9] including 293 patients with SAB reported an adjusted odds ratio of 2.4 (95% CI, 1.2–4.7) for 30-day mortality among patients with DM compared to those without DM. A cohort study including 424 patients with SAB from New Zealand [10] reported an age- and sex-adjusted relative risk of 1.5 (95% CI, 1.0–2.4) for patients with DM compared to patients without. However, both studies were limited by relatively small patient numbers (n<500), selected study populations, and a lack of detailed data on DM. In a clinical trial designed to evaluate treatment of infective endocarditis due to *S*. *aureus*, Kanafani et al. [31] observed an overall mortality at 6 weeks of 22.1% in patients with DM compared to 11.4% in patients without DM. These estimates, however, were based on a subgroup analysis including a limited number of DM patients with concurrent infective endocarditis (n = 86), which may in part explain the observed difference in outcome. In addition, Kanafani et al. did not distinguish between CA-SAB and hospital-acquired SAB; indeed, investigators did not discern between patients with CA-SAB and HCA-SAB in any of these three previous studies [9–10,31].

Several explanations are possible for the observed lack of increased mortality in patients with DM. In these patients, the inflammatory response to an acute infectious challenge is impeded [8,31], which might influence SAB prognosis in a positive direction. Nevertheless, in our study, we observed almost similar levels of C-reactive protein (CRP) on admission among patients with and without DM. Furthermore, persons with DM may interact with the healthcare system more frequently than do those in the general population, and physicians may be more likely to admit a patient with DM on suspicion of infection compared to patients without DM. Consequently, time to blood culture sampling and initiation of antibiotic therapy could have been shorter in patients with DM. Such surveillance bias would lead to underestimation of DM-related mortality; however, the proportions of patients classified as HCA-SAB did not differ notably among the two groups, the CRP levels on admission were comparable, and patients with DM were not more likely to have received preadmission antibiotic treatment than patients without DM. These factors could counter but do not preclude bias associated with the clinical management of patients with DM in our setting. The majority of patients in our cohort were characterized by advanced age and several concurrent comorbid conditions. These characteristics may reflect that the high mortality associated with SAB is more dependent on the combined burden of age and comorbidities and less so on individual comorbid conditions such as DM and characteristics (e.g., glycaemic control) and complications associated with DM.

Our study’s strengths include its considerable size, the use of routinely recorded clinical data, and the population-based design with information at the individual level and virtually complete follow-up for death. In contrast to previous studies, in the current work, patients with DM were identified via a previously validated algorithm [24], and detailed data on characteristics of DM patients were available. Our study also has a number of limitations. Misclassification of patients with DM would bias our results towards the null, and although our ascertainment of DM was based on three separate comprehensive registries, we cannot entirely preclude that some patients with DM may have been missed (e.g., patients treated with diet and lifestyle changes alone). Furthermore, we cannot rule out that some cases of SAB were missed if the patient had received preadmission antibiotic treatment, if the patient had been hospitalized outside of the catchment area, or had died before blood culture sampling [32]. If either of these outcomes pertained to patients with DM in particular, mortality in this group might have been underestimated. Moreover, the DNPR does not include information on comorbidity (including diabetes and diabetes complications) diagnosed in the primary care sector. Due to the historical design or our study, we lacked data on infective foci, including central venous catheters and other vascular access devices, which have been associated with SAB outcome in previous studies [3]. A difference in distribution of foci among patients with and without DM could have biased our estimates. Socioeconomic factors influence prognosis in patients with SAB [3,33], but we did not have access to data on educational level and personal income. Nevertheless, based on the equal and cost-free access to healthcare in Denmark, we do not expect these potential confounders to be unevenly distributed among patients with and without DM.

In conclusion, CA-SAB patients with DM did not experience higher 30-day all-cause mortality compared to patients without DM, and we observed no substantial differences in mortality across subsets of patients. However, the considerable 30-day mortality identified in our study emphasizes the need for continued research on prognostic factors of SAB to facilitate improved management and patient outcomes.

## Supporting Information

S1 Appendix Identification and susceptibility testing of *S*.*aureus* isolates. (PDF)

S2 Appendix Codes for diagnoses, procedures, medication and blood tests. (PDF)

S1 Table Crude and adjusted mortality within 30 days comparing incident *S*.*aureus* bacteraemia patients with and without diabetes, stratified by sex, age, marital status and modified Charlson Comorbidity Index score. Analyses restricted to patients with community-acquired *S*. *aureus* bacteraemia (n = 1523).
(PDF)

## References

[1] Thwaites GE, Edgeworth JD, Gkrania-Klotsas E, Kirby A, Tilley R, Estée Török M, et al Clinical management of Staphylococcus aureus bacteraemia. Lancet Infect Dis 2011; 11:208–22. 10.1016/S1473-3099(10)70285-1 21371655

[2] Kern WV. Management of *Staphylococcus aureus* bacteremia and endocarditis: progress and challenges. Curr Opin Infect Dis 2010; 23:346–358. 10.1097/QCO.0b013e32833bcc8a 20592532

[3] van Hal SJ, Jensen SO, Vaska VL, Espedido BA, Paterson DL, Gosbell IB. Predictors or mortality in Staphylococcus aureus bacteremia. Clin Microbiol Rev 2012; 25:362–86. 10.1128/CMR.05022-11 22491776 PMC3346297

[4] Lesens O, Methlin C, Hansmann Y, Remy V, Martinot M, Bergin C et al Role of comorbidity in mortality related to Staphylococcus aureus bacteremia: A prospective study using the Charlson Weighted Index of comorbidity. Infect Control Hosp Epidemiol 2003; 24:890–6. 14700403 10.1086/502156

[5] Mejer N, Westh H, Schønheyder HC, Jensen AG, Larsen AR, Skov R, et al Stable incidence and continued improvement in short term mortality of Staphylococcus aureus bacteraemia between 1995 and 2008. BMC Infect Dis 2012; 12:260 10.1186/1471-2334-12-260 23075215 PMC3507819

[6] Guariguata L, Whiting DR, Hambleton I, Beagley J, Linnenkamp U, Shaw JE. Global estimates of diabetes prevalence for 2013 and projections for 2035. Diabetes Res Clin Pract 2014; 103:161–75 24630390 10.1016/j.diabres.2013.11.002

[7] Whiting DR, Guariguata L, Weil C, Shaw J. IDF diabetes atlas: global estimates of the prevalence of diabetes for 2011 and 2030. Diabetes Res Clin Pract 2011; 94:311–21. 10.1016/j.diabres.2011.10.029 22079683

[8] Thomsen RW, Mor A. Diabetes and risk of community-acquired respiratory tract infections, urinary tract infections, and bacteremia. The Open Infectious Diseases Journal 2012; 6:27–39.

[9] Mylotte JM, Tayara A. *Staphylococcus aureus* bacteremia: predictors of 30-day mortality in a large cohort. Clin Infect Dis 2000; 31:1170–4. 11073748 10.1086/317421

[10] Hill PC, Birch M, Chambers S, Drinkovic D, Ellis-Pegler RB, Everts R, et al Prospective study of 424 cases of *Staphylococcus aureus* bacteraemia: determination of factors affecting incidence and mortality. Intern Med J 2001; 31:97–103. 11480485

[11] Leibovici L, Samra Z, Konisberger H, Kalter-Leibovici, Pitlik SD, Drucker M. Bacteremia in adult diabetic patients. Diabetes Care 1991; 14:89–94. 2060428 10.2337/diacare.14.2.89

[12] Laupland KB, Church DL, Mucenski M, Sutherland LR, Davies HD. Population-based study of the epidemiology of and the risk factors for invasive Staphylococcus aureus infections. J Infect Dis 2003; 187:1452–9. 12717627 10.1086/374621

[13] Kaech C, Elzi L, Sendi P, Frei R, Laifer G, Bassetti S, et al Course and outcome of Staphylococcus aureus bacteremia: a retrospective analysis of 308 episodes in a Swiss tertiary-care centre. Clin Microbiol Infect 2006; 12:345–352. 16524411 10.1111/j.1469-0691.2005.01359.x

[14] Kaasch AJ, Barlow G, Edgeworth JD, Fowler VG, Hellmich M, Hopkins S, et al Staphylococcus aureus bloodstream infection: A pooled analysis of five prospective, observational studies. J infect 2014; 68:242–251. 10.1016/j.jinf.2013.10.015 24247070 PMC4136490

[15] Vallés J, Calbo E, Anoro E, Fontanals D, Xercavins M, Espejo E, et al Bloodstream infections in adults: Importance of healthcare-associated infections. J Infect 2008;56:27–34. 18022242 10.1016/j.jinf.2007.10.001

[16] Lenz R, Leal JR, Church DL, Gregson DB, Ross T, Laupland KB. The distinct category of healthcare associated bloodstream infections. BMC Infect Dis 2012;12:85 10.1186/1471-2334-12-85 22487002 PMC3364909

[17] Shorr A, Tabak YP, Killian AD, Gupta V, Liu LZ, Kollef MH. Healthcare-associated bloodstream infections: A distinct entity? Insights from a large U.S. database. Crit Care Med 2006;10:2588–95. 10.1097/01.CCM.0000239121.09533.09 16915117

[18] Pedersen CB. The Danish Civil Registration System. Scan J Public Health 2011; 39:22–25. 10.1177/1403494810387965 21775345

[19] Schmidt M, Pedersen L, Sørensen HT. The Danish Civil Registration System as a tool in epidemiology. Eur J Epidemiol 2014; 29:541–549. 10.1007/s10654-014-9930-3 24965263

[20] Wiese L, Mejer N, Schønheyder HC, Westh H, Jensen AG, Larsen AG, et al A nationwide study of comorbidity and risk of reinfection after Staphylococcus aureus bacteraemia. J Infect 2013;67:199–205. 10.1016/j.jinf.2013.04.018 23664855

[21] Smit J, Søgaard M, Schønheyder H, Nielsen H, Frøslev T, Thomsen RW. Classification of healthcare-associated Staphylococcus aureus bacteremia: Influence of different definitions on prevalence, patient characteristics, and outcome. Infect Control Hosp Epidemiol 2016;37(2):208–211 10.1017/ice.2015.259 26503397

[22] Lynge E, Sandegaard JL, Rebolj M. The Danish National Patient Register. Scand J Public Health 2011; 39:30–33. 10.1177/1403494811401482 21775347

[23] Schmidt M, Schmidt SAJ, Sandegaard JL, Ehrenstein V, Pedersen L, Sørensen HT. The Danish National Patient Registry: a review of content, data quality, and research potential. Clin Epidemiol 2015;7:449–490. 10.2147/CLEP.S91125 26604824 PMC4655913

[24] Thomsen RW, Hundborg HH, Lervang HH, Johnsen SP, Sørensen HT, Schønheyder HC. Diabetes and outcome of community- acquired pneumococcal bacteremia. Diabetes Care 2004; 27:70–6. 14693969 10.2337/diacare.27.1.70

[25] Grann AF, Erichsen R, Nielsen AG, Frøslev T, Thomsen RW. Existing data sources for clinical epidemiology: The clinical laboratory information system (LABKA) research database at Aarhus University, Denmark. Clin Epidemiol 2011; 1:133 10.2147/CLEP.S17901 PMC3072155 21487452

[26] Ehrenstein V, Antonsen S, Pedersen L. Existing data sources for clinical epidemiology: Aarhus University Prescription Database. Clin Epidemiol 2010; 2:273–279. 10.2147/CLEP.S13458 21152254 PMC2998817

[27] Levey AS, Coresh J, Greene T, Stevens LA, Zhang YL, Hendriksen S, et al Using standardized serum creatinine values in the modification of diet in renal disease study equation for estimating glomerular filtration rate. Ann Intern Med 2006; 145:247–254. 16908915 10.7326/0003-4819-145-4-200608150-00004

[28] Charlson M, Pompei P, Ales KL. A new method of classifying prognostic comorbidity in longitudinal studies: Development and validation. J Chron Dis 1987; 40:373–83. 3558716 10.1016/0021-9681(87)90171-8

[29] Thygesen SK, Christiansen CF, Christensen S, Lash T, Sørensen HT. The predictive value of ICD-10 diagnostic coding used to assess Charlson comorbidity index conditions in the population-based Danish National Registry of Patients. BMC Med Res Methodol 2011;11:83 10.1186/1471-2288-11-83 21619668 PMC3125388

[30] López-Cortes LE, Gálvez-Acebal J, del Toro MD, Velasco C, de Cueto M, Caballero FJ, et al Effect of statin therapy in the outcome of blood stream infections due to Staphylococcus aureus. A prospective cohort study. PLoS One 2013; 8:e82958 10.1371/journal.pone.0082958 24376617 PMC3871563

[31] Kanafani Z a, Kourany WM, Fowler VG, Levine DP, Vigliani GA, Campion M, et al Clinical characteristics and outcomes of diabetic patients with Staphylococcus aureus bacteremia and endocarditis. Eur J Clin Microbiol Infect Dis 2009;28:1477–82. 10.1007/s10096-009-0808-3 19730900

[32] Søgaard M, Engebjerg MC, Lundbye-Christensen S, Schønheyder HC. Changes in blood culture methodology have an impact on time trends of bacteraemia: a 26-year regional study. Epidemiol Infect 2011;139:772–776. 10.1017/S095026881000169X 20619078

[33] Koch K, Nørgaard M, Schønheyder HC, Thomsen RW, Søgaard M, for the Danish Collaborative Bacteremia Network (DACOBAN). Effect of socioeconomic status in mortality after bacteremia in working-age patients. A Danish population-based cohort study. PLoS One 2013;8:e70082 10.1371/journal.pone.0070082 23936145 PMC3723741

