# Supplementary material for: Outcome of Community-Acquired Staphylococcus aureus Bacteraemia in Patients with Diabetes: A Historical Population-Based Cohort Study
Source: PLoS One. 2016 Apr 15;11(4):e0153766. doi: 10.1371/journal.pone.0153766 (PMC4833306; doi:10.1371/journal.pone.0153766)
Supplement: S1 Appendix — (PDF) [file pone.0153766.s001.pdf]

S1 Appendix

Identification and susceptibility testing of *S.aureus* isolates

Setting

During the study period a reform of local government merged four counties into two heath regions: Central Denmark Region and North Denmark Region, collectively referred to as Northern Denmark. Three departments of clinical microbiology served Central Denmark Region and were situated in Aarhus (Aarhus University Hospital), Viborg (Regional Hospital of Viborg) and Herning (Regional Hospital West Jutland). North Denmark Region was served by one department of clinical microbiology at Aalborg University Hospital, Aalborg.

The total number of beds in non-psychiatric wards was 5.528 in 2000 and 4.058 in 2011.

Number of hospitals in the two health regions and their predecessors

	2000	2011
Central Denmark Region	17	5
North Denmark Region	7	4

Blood cultures

Blood cultures were ordered by the attending physician and blood samples were drawn by trained biotechnicians. The BacT/Alert system (bioMérieux, Marcy l'Etoil, France) was used throughout the study period at all hospital sites. Recommendations differed between the two health regions: In Central Denmark Region a standard blood culture for adults comprised two sets with two bottles each (one aerobic and one anaerobic bottle), whereas the standard in North Denmark Region was one set with three bottles (two aerobic and one anaerobic bottle).

Identification and susceptibility testing of *Staphylococcus aureus*

S. aureus was identified by horse plasma tube coagulase test or an equivalent commercial latex agglutination test. All blood culture isolates were referred to the Staphylococcal Reference Laboratory at Statens Serum Institut (Copenhagen) for national surveillance which included phage typing up to 2006 and *spa*-typing thereafter.[1-2]

Susceptibility testing was undertaken locally by disk diffusion and confirmatory testing was performed at Statens Serum Institut.[3-5] Screening for methicillin resistance varied between departments 2000-2002, but in 2003 the cefoxitin disk diffusion test was implemented both locally and at Statens Serum Institut.[4-5] Detection of the *mecA* gene cassette was done by either in-house polymerase chain reaction (PCR) or the EVIGENE™ hybridization test.[2,7]

Methicillin-resistant *S. aureus* (MRSA) became notifiable in Denmark in 2006. Due to the low prevalence of methicillin-resistance extra precautions were taken in this study by cross-referencing local data with the Danish national *S. aureus* bacteremia database at Statens Serum Institut.

References

1. DANMAP 2006 - Use of antimicrobial agents and occurrence of antimicrobial resistance in bacteria from food animals, foods and humans in Denmark. ISSN 1600-2032. http://www.danmap.org/~/media/Projekt%20sites/Danmap/DANMAP%20reports/Danmap_2003.ashx (Accessed June 1 2015).
2. Larsen AR, Stegger M, Sørum M. spa typing directly from a mecA, spa and pvl multiplex PCR assay-a cost-effective improvement for methicillin-resistant Staphylococcus aureus surveillance. Clin Microbiol Infect. 2008 Jun;14(6):611-4.
3. DANMAP 2000 – Consumption of antimicrobial agents and occurrence of antimicrobial resistance in bacteria from food animals, foods and humans in Denmark. ISSN 1600-2032. http://www.danmap.org/~/media/Projekt%20sites/Danmap/DANMAP%20reports/Danmap_2000.ashx (Accessed June 1 2015).
4. DANMAP 2003 - Use of antimicrobial agents and occurrence of antimicrobial resistance in bacteria from food animals, foods and humans in Denmark. ISSN 1600-2032. http://www.danmap.org/~/media/Projekt%20sites/Danmap/DANMAP%20reports/Danmap_2003.ashx (accessed June 1 2015)
5. Skov R, Smyth R, Clausen M, Larsen AR, Frimodt-Møller N, Olsson-Liljequist B, Kahlmeter G. Evaluation of a cefoxitin 30 microg disc on Iso-Sensitest agar for detection of methicillin-resistant Staphylococcus aureus. J Antimicrob Chemother. 2003 Aug;52(2):204-7. Epub 2003 Jul 1. PubMed PMID: 12837728.

6. Skov RL, Pallesen LV, Poulsen RL, Espersen F. Evaluation of a new 3-h hybridization method for detecting the mecA gene in Staphylococcus aureus and comparison with existing genotypic and phenotypic susceptibility testing methods. J Antimicrob Chemother. 1999 Apr;43(4):467-75.
