# Supplementary material for: Outcome of Community-Acquired Staphylococcus aureus Bacteraemia in Patients with Diabetes: A Historical Population-Based Cohort Study
Source: PLoS One. 2016 Apr 15;11(4):e0153766. doi: 10.1371/journal.pone.0153766 (PMC4833306; doi:10.1371/journal.pone.0153766)
Supplement: S2 Appendix — (PDF) [file pone.0153766.s002.pdf]

S2 Appendix

Codes for diagnoses, procedures, medication and blood tests

Formula for estimation of glomerular filtration rates (eGFR)

Preadmission comorbid conditions. Diagnoses codes are according to the 8th and 10th revision of the International Classification of Diseases, ICD-8 and ICD-10

**Condition**	**ICD-8**	**ICD-10**
Diabetes	249.00, 249.06, 249.07, 249.09, 250.00, 250.06, 250.07, 250.09	E10-E14. O24 (except O24.4), G63.2, H36.0, N08.3
Myocardial infarction	410	I21-I23
Congestive heart failure	427.09, 427.10, 427.11, 427.19, 428.99, 782.49	I50, I11.0, I13.0, I13.2
Peripheral vascular disease	440, 441, 442, 443, 444, 445	I70, I71, I72, I73, I74, I77
Cerebrovascular disease	430-438	I60-I69, G45, G46
Dementia	290.09-290.19, 293.09	F00-F03, F05.1, G30
Chronic pulmonary disease	490-493, 515-518	J40-J47, J60-J67, J68.4, J70.1, J70.3, J84.1, J92.0, J96.1, J98.2, J98.3
Connective tissue disease	712, 716, 734, 446, 135.99	M05, M06, M08, M09, M30, M31, M32, M33, M34, M35, M36, D86
Ulcer disease	530.91, 530.98, 531-534	K22.1, K25-K28

Mild liver disease	571, 573.01, 573.04	B18, K70.0-K70.3, K70.9, K71, K73, K74, K76.0
Hemiplegia	344	G81, G82
Moderate to severe renal disease	403, 404, 580-583, 584, 590.09, 593.19, 753.10-753.19, 792	I12, I13, N00-N05, N07, N11, N14, N17-N19, Q61
Any tumor	140-194	C00-C75
Leukemia	204-207	C91-C95
Lymphoma	200-203, 275.59	C81-C85, C88, C90, C96
Moderate to severe liver disease	070.00, 070.02, 070.04, 070.06, 070.08, 573.00, 456.00-456.09	B15.0, B16.0, B16.2, B19.0, K70.4, K72, K76.6, I85
Metastatic solid tumor	195-198, 199	C76-C80
AIDS	079.83	B21-B24
Hypertension	400-404	I10-I13
Osteoporosis	723.09	M80-M82
Conditions related to alcohol abuse	291.09-291.99, 303.09-303.29, 303.91-303.99	F10, K86.0, Z72.1. T51, K29.2, G62.1, G31.2, I42.6, K70
Conditions related to drug abuse	304.09-304.99	F11-F16, F18-F19, T40
Microvacular complications:		
• Diabetic retinopathy	250.01, 249.01	H36.0, E10.3, E11.3, E12.3, E13.3, E14.3, H28.0, H33.4, H45.0

• Diabetic nephropathy	250.02, 249.02	N08.3, E10.2, E11.2, E12.2, E13.2, E14.2
• Diabetic neuropathy	250.03, 249.03	E10.4, E11.4, E14.4, G59.0, G63.2
Macrovascular complications:		
• Ischemic heart disease including atherosclerosis	410.09-414.99	I20-I25
• Stroke, transient cerebral ischemia and cerebrovascular disease	432.00-437.99	G45, I61, I63-I66, I67.2, I67.8-I67.9, I69.1, I69.3-I69.8
• Peripheral arterial disease	440.09-440.29	I70.2, I74.2-I74.5<br>I73.9A, I73.9B, I73.9C<br>E10.5, E11.5

Procedures. Codes regarding dialysis are according to Danish Treatment codes and according to the 10th revision of the International Classification of Diseases, ICD-10.

	**Danish treatment codes**	**ICD-10**
**Dialysis**	98300, 94340, 94350<br>BJFD0, BJFD2	Z99.2, Z49, Z49.2, BJFD
**Surgery**	All surgical codes (K-codes) in the Nordic Medico-Statistical Committee (NOMESCO) Classification of Surgical Procedures.	

Medication codes are according to the Anatomical Therapeutic Classification (ATC)	
**Type of medication**	**ATC codes**
Antidiabetic medication:	
All antidiabetic medication	A10A, A10B
• Insulin	A10A
• Metformin	A10BA02
• Sulfonylureas	A10BB, A10BC
• Any other antidiabetic drugs	A10 without A10A, A10BA02, A10BB, A10BC
Immunosuppressive therapy	L01, L04
Systemic antibiotic therapy	J01
ACE inhibitors	C09
Beta-blockers	C07
Acetylsalicylic acid	B01AC06
Other thrombocyte function inhibitors	B01AC04, B01AC07, B01AC30
Statins or lipid lowering agents	C10AA, C10B, B04AB, C10AB, C10AC, C10AD, C10AX

Blood tests according to local analysis codes and Nomenclature for Properties and Units (NPU)-codes	
**Blood test**	**Local analysis and NPU-codes**
C-reactive protein	DNK05027, ASS00653, ASS00010, AAA94016, ASS00080, 1314609, ASS00080, 5027, 1314610, 1414610, 16, 113039, 1810752, 1311097, 1314610, 1314612, 1314611, 1414609, 1314614,1414614, 19748, 1423, 1314612<br><br>NPU19748, NPU01423
Creatinine	ASS00356, ASS00354, ASS00355, 11026, 1511235,1511236, 1610154, 1710301,1711807, 1811807, 1817156, 18016,1155,1311235, 1411235, 38927, 4998, 1611807<br><br>NPU18016, NPU01807, NPU18105, NPU04998

HbA1c	AAB00091, AAA00740, AAB00061, AAB00092, 12155<br><br>NPU03835, NPU02307, NPU27300
Glucose	ASS00203, ASS00204, DNK35842, 352, 1460, 161, 1462, 1311500, 1311551, 1311597, 132600, 1321000, 1411500, 1411551, 1411597, 8792, 2195, 35842, 112069, 112195, 113016, 114325, 1511500, 1511631, 1511700, 161034, 1610823, 1616560, 161635, 1710343, 1712188, 1812188, 1812195, 1817225, 1817426,140600, AAA00317, AAA00320, AAA00321, AAA00230, AAA00308, AAA00311, AAA00314<br><br>NPU02193, NPU02195, NPU08509, NPU08972, NPU22068, NPU22069, NPU02187, NPU02192, NPU04093, NPU22095, NPU08869-NPU08916, NPU22069-NPU22089, NPU22099-NPU22126, NPU08519-NPU08567, NPU21531, NPU21533

Formula for estimation of glomerular filtration rates (eGFR)

GFR (mL/min/1.73 m2) = 175 x (Scr/88.4)-1.154 x (Age)-0.203 x (0.742 if female) x (1.212 if African American) (SI units).

Scr = serum creatinine.
