# Supplementary material for: Outcome of Community-Acquired Staphylococcus aureus Bacteraemia in Patients with Diabetes: A Historical Population-Based Cohort Study
Source: PLoS One. 2016 Apr 15;11(4):e0153766. doi: 10.1371/journal.pone.0153766 (PMC4833306; doi:10.1371/journal.pone.0153766)
Supplement: S1 Table — Analyses restricted to patients with community-acquired S. aureus bacteraemia (n = 1523). (PDF) [file pone.0153766.s003.pdf]

S1 Table. Crude and adjusted mortality within 30 days comparing incident *S.aureus* bacteremia patients with and without diabetes, stratified by sex, age, marital status and modified Charlson Comorbidity Index score. Analyses restricted to patients with community-acquired *S. aureus* bacteremia (n=1523).

	Patients without diabetes	Patients with diabetes	
	30-day mortality (95% CI)	30-day mortality (95% CI)	Adj. MRR[1] (95% CI)
Overall	24.8 (22.4-27.5)	30.6 (26.3-35.4)	1.13 (0.90-1.41)
Sex			
Male	22.2 (19.3-25.5)	24.9 (20.1-30.6)	0.98 (0.72-1.32)
Female	29.0 (25.0-33.5)	41.5 (33.7-50.3)	1.40 (1.00-1.96)
Age			
15-39	3.3 (1.3-8.6)	10.0 (14.7-52.7)	35.07 (1.06-1156.99)
40-59	12.9 (9.3-17.8)	21.0 (12.8-33.4)	2.03 (1.01-4.06)
60-79	25.3 (21.5-29.6)	24.0 (18.7-30.5)	0.83 (0.57-1.19)
80+	41.8 (35.6-47.4)	48.3 (39.8-57.6)	1.31 (0.95-1.81)
Marital status			
Married	19.4 (16.1-23.3)	22.9 (17.2-30.1)	1.04 (0.70-1.56)
Divorced or widowed	35.8 (31.4-40.6)	36.7 (30.1-44.3)	1.08 (0.80-1.46)
Never married	16.3 (12.2-21.7)	34.0 (22.9-48.4)	2.13 (1.16-3.92)

Modified Charlson Comorbidity Index			
Low (0)	19.4 (16.3-23.1)	21.2 (14.9-29.7)	1.05 (0.67-1.66)
Intermediate (1-2)	29.0 (24.9-33.6)	29.7 (23.5-37.1)	1.02 (0.73-1.42)
High (3+)	31.4 (25.0-38.9)	42.7 (33.8-52.8)	1.39 (0.90-2.13)

Reference group: patients without diabetes. CI, confidence interval.

[1]Adj. MRR: Mortality rate ratio adjusted for age, gender, marital status, conditions included in the modified Charlson Comorbidity Index, hypertension, alcohol related conditions, any previous statin use prior to admission, and antibiotic therapy 30 days prior to admission.
